# Construction of a BAC library and mapping BAC clones to the linkage map of Barramundi, *Lates calcarifer*

**DOI:** 10.1186/1471-2164-9-139

**Published:** 2008-03-25

**Authors:** Chun Ming Wang, Loong Chueng Lo, Felicia Feng, Ping Gong, Jian Li, Ze Yuan Zhu, Grace Lin, Gen Hua Yue

**Affiliations:** 1 Molecular Population Genetics Group, Temasek Life Sciences Laboratory, 1 Research Link, National University of Singapore, 117604, Singapore

## Abstract

**Background:**

Barramundi (*Lates calcarifer*) is an important farmed marine food fish species. Its first generation linkage map has been applied to map QTL for growth traits. To identify genes located in QTL responsible for specific traits, genomic large insert libraries are of crucial importance. We reported herein a bacterial artificial chromosome (BAC) library and the mapping of BAC clones to the linkage map.

**Results:**

This BAC library consisted of 49,152 clones with an average insert size of 98 kb, representing 6.9-fold haploid genome coverage. Screening the library with 24 microsatellites and 15 ESTs/genes demonstrated that the library had good genome coverage. In addition, 62 novel microsatellites each isolated from 62 BAC clones were mapped onto the first generation linkage map. A total of 86 BAC clones were anchored on the linkage map with at least one BAC clone on each linkage group.

**Conclusion:**

We have constructed the first BAC library for *L. calcarifer *and mapped 86 BAC clones to the first generation linkage map. This BAC library and the improved linkage map with 302 DNA markers not only supply an indispensable tool to the integration of physical and linkage maps, the fine mapping of QTL and map based cloning genes located in QTL of commercial importance, but also contribute to comparative genomic studies and eventually whole genome sequencing.

## Background

Barramundi (*Lates calcarifer*), also called Asian seabass or the giant sea perch, belonging to the family Latidae is widely distributed in the coast and freshwater of the tropical Indo-west Pacific, from the Persian Gulf to India and Northern Australia [1,2]. Because of good meat quality and relatively high market value of *L. calcarifer*, it has become an attractive commodity of both large and small-scale aquaculture enterprises. It is commercially cultivated in Thailand, Malaysia, Singapore, Indonesia, Hong Kong, China and Australia in both brackishwater and freshwater ponds, as well as in cages in coastal waters. The global annual production of *L. calcarifer *was 400,000 MT according to FAO statistics. However, detailed breeding programs for genetic improvement of Asian seabass are still quite rare [3]. Identification of genomic regions and genes responsible for economically important traits could facilitate genetic improvement through marker-assisted selection [4], which is of importance for future aquaculture of *L. calcarifer*.

Linkage and physical maps are indispensable tools needed to identify genomic regions responsible for traits of interest. The genome of *L. calcarifer *is very compact (only 700 Mb) consisting of 24 chromosome pairs [5]. The first linkage map for *L. calcarifer *containing 240 microsatellite markers and genes on 24 linkage groups [6] was applied to mapping QTL for growth traits [7]. Libraries with large genomic DNA inserts are essential for physical mapping and positional cloning, particularly for higher eukaryotes [8]. The BAC (bacterial artificial chromosome) cloning system has become an invaluable tool in genomics studies because of its ability to stably maintain large DNA fragments and its ease of manipulation [9]. Genomic inserts in BAC clones have been shown to be very stable in *E. coli *and thus serve as ideal templates in generating whole-genome physical maps by DNA fingerprinting, developing sequence-tagged connectors and shotgun sequencing [10-12]. These features make the BAC cloning system a popular choice for high-throughput genomics studies [13]. BAC libraries have been developed for many economically important animal species such as cattle [14], pig [15], and sheep [16] and the highly endangered giant panda [17]. Only currently, BAC libraries were produced for some commercially important fish species such as salmon [18], catfish [12], rainbow trout, carp and tilapia [19].

Here, we describe the construction and characterization of a BAC library covering 6.9 times *L. calcarifer *haploid genome and mapping of 86 BAC clones to the linkage map. The BAC library and the improved linkage map of *L. calcarifer *will facilitate the integration of physical and linkage maps, fine mapping of QTL and identification of genes located in QTL of interest, maker-assisted selection and genome research.

## Results

### Library construction

A BAC library of *L. calcarifer *was constructed using the *Hin*dIII cloning site in commercially prepared pCC1BAC vector (Epicentre, MD, USA). The BAC library consisted of a total of 49,152 clones, which were manually arrayed into 128 384-well plates.

### Insert size distribution

**Figure 1:**
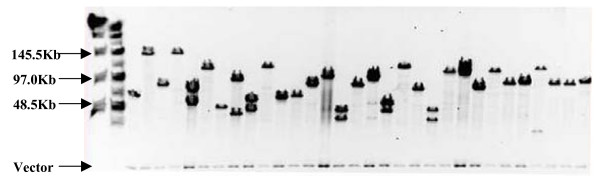
**DNA analysis of 31 random BAC clones from the *L. calcarifer Hin*dIII BAC library by pulse-field gel electrophoresis**. DNA samples digested with *Not*I were separated on 1% agarose gel in 0.5 × TBE buffer for 16 h under the following conditions: ramp pulse time of 5–15 s at 6 V/cm, temperature at 14°C. Markers used are Lambda Ladder PFG Marker (outside lanes) and MidRange II PFG Marker (NEB, SG, Singapore). The 8 kb common band is the pCC1BAC Vector (Epicentre, WI, USA).

**Figure 2:**
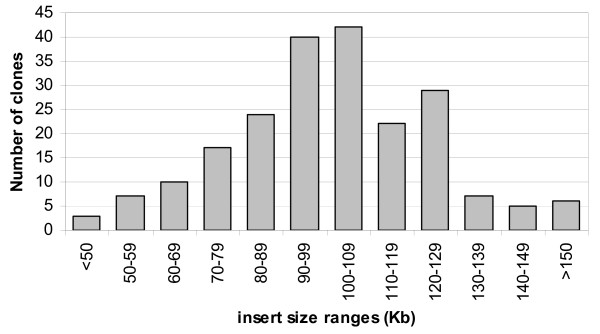
**Insert size distribution of 212 *L. calcarifer *BAC clones**. DNA samples of the 212 clones randomly picked from the *L. calcarifer Hin*dIII BAC library were analyzed and grouped. Results indicate that the average insert size is 98 kb with over 80% of the clones > 80 kb.

To examine the quality of the BAC library, the sizes of 212 BAC clones randomly picked from the library were determined. All the 212 clones contained inserts. The insert size distribution of these 212 clones is shown in Figure 1 and 2. The average insert size was 98 kb, ranging from 45 to 200 kb. The insert size of over 80% of the BAC clones in this library was larger than 80 kb, and the insert size of 50% clones was smaller than 100 kb. This BAC library provides 6.9 time haploid genome equivalent based on a genome size of 700 Mb [6].

### BAC library screening

**Figure 3:**
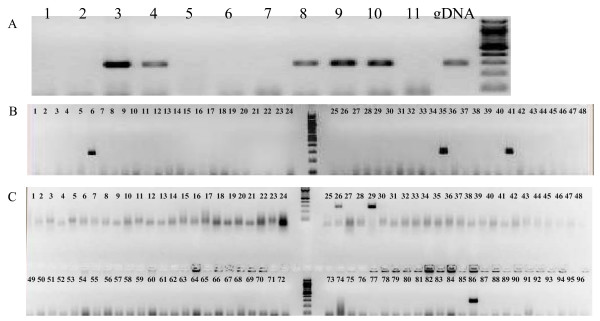
**Hierarchical PCR screening of the superpools and pools of the BAC library of *L. calcarifer***. **A **First round PCR screening in 11 superpools representing the entire library or 128 384-well microtiter plates. Lanes 1–11: superpools 1–11 and lane 12: genomic DNA as positive control. Each superpool contains DNA of 12 plates or 4,608 individual BAC clones. In five superpools (3, 4, 8, 9 and 10), PCR product was amplified by the marker Lca064. **B **Second round PCR screening in 48 pools from the superpool number 3. Three pools (6, 35 and 41) showed a signal amplified by the marker Lca064. **C **Third round PCR screening in a 96-well plate from the pool number 6. Three positive clones (26, 29 and 86) were detected in the plate by the marker Lca064.

To further assess the quality of this BAC library, 24 microsatellite markers randomly selected from each of the 24 linkage groups were used for hierarchical screening. PCR-screenings with the 24 microsatellite markers resulted in the number of positive clones varying from 3 to 15 with an average of 6.6 (Table 1) (see example of the PCR screening in Figure 3). PCR screening with these 15 ESTs/genes (PVALB-1, 5-HT, PROL-A, 14KDA-AP, AMY-A, MX, AP, LECT2, LYSO-G, IGF-1, TUB1A, TUB2B, GT7, CYP19A2 and AFPII) revealed that the number of positive BAC clones varied from 3 to 14 with an average of 7.3 (Table 2). The actual average number of positive BAC clones detected by screening with microsatellites and ESTs/genes was near to theoretically calculated number of genome coverage (i.e. 6.9 time coverage of the haploid genome). At least 3 positive BAC clones for each of the ESTs/genes were identified from the library.

**Table 1:** Screening of the BAC library with 24 microsatellites located on each of the 24 linkage groups of *L. calcarifer*

Linkage group	Locus	GenBank accession no	Forward primer (5'-3')	Reverse primer (5'-3')	PCR Ta (°C)	Positive clone number
LG1	Lca318	DQ290175	TCCCACCCCAGTCCAGAAA	TACCAGAGCCTGAAACACAGTAGG	55	6
LG2	Lca064	AY998856	AGGCATATGCACCTCACAAGAGTG	CCCACGGTTTATTTATCTGTCATTATC	55	15
LG3	Lca137	DQ290039	CGCCTTAAATCTCTACGCTCTGG	TCGCATGCTGTAATTAAGGTGGTA	55	5
LG4	Lca171	DQ290065	ATTGCGTTACCAAGAGGTGAA	TGTCTTTGAAGGCTGAAAACTG	55	8
LG5	Lca098	AY998880	CAAAGGGGCCACTGCACATAAT	CTCCAGCTCACCCAGGTTCACT	55	5
LG6	Lca062	AY998854	AGGATGGCACGCTGAAACTATCG	ATAAGCTTGTACAGGGGCTGAGTGC	55	3
LG7	Lca130	DQ290035	GAGGCTCCCAATCCCAACAA	GGAGGCAGACGAGGAAGGAA	55	13
LG8	Lca086	AY998873	AAATGGCCTTCCTGTCCCTTCAG	GTGTTCCCTTGTTCTGCCACAGTG	55	4
LG9	Lca301	DQ290166	GCCAGTGTGAGGGACAGAGA	GGGCCTTGTTTTGCTTTTG	55	9
LG10	Lca002	AF007943	GCCGCTTGTTTACCAGTAAA	TCCATTTGAGGATTAACAGC	55	5
LG11	Lca058	AY998850	AAACAGGCAGCCAGATAGACAGAG	AAGAGGTGGTGGGACTAATTTGAGA	55	13
LG12	Lca074	AY998863	CATCATTTACACTCTGTTTGCCTCAT	GACAGACAGGTGTTTTAGCCTATTTG	55	6
LG13	Lca253	DQ290129	TGGGGACTTGACTTCCTTTTATG	TACCGAGGTTGGATGGTTTTCT	55	3
LG14	Lca147	DQ290047	TGCCCCTAATGTATTCTTTCCACT	GCTCCCACCTCTCATTCATTATTC	55	5
LG15	Lca069	AY998859	GCCTTTCTGTTTTCTGATTTATCTTCAT	AACACCCCGAAATACTGCTACTACAG	55	4
LG16	Lca367	DQ290206	TGTATTACAATGCCCGTGGTCA	TTAAGCCTTTGGTGTCTCAGTGTG	55	10
LG17	Lca021	AF404083	GTGCCACCTGCCTGACC	GCCATGACTGATTGCTGAGA	55	4
LG18	Lca193	DQ290082	CCTCTGCCTTTTCATCTATATTGC	CACATCGCACAAATGGACTGA	55	9
LG19	Lca220	DQ290104	ATGGCTGTGAAAAGACTGGTATCT	CGCCCCTCACTCAACAGAG	55	5
LG20	Lca181	DQ290073	CACTGGGTGGCGTTTGTATTAGC	CAAGAATTGGGATTTTGCTGTGC	55	8
LG21	Lca255	DQ290131	AGAGACACTTTATACGGGGACATC	GTAAACTGAAGCAAGCCAAACCT	55	7
LG22	Lca040	AF404099	TGAGGAAGCATCAGCTGTAATCA	CAGGACGCAAACACTGAAAT	55	3
LG23	Lca411	DQ290221	GTGGTGCAGCGGTTGCTCTC	CCGACTCATGCTGCTTTTCGTAAT	55	5
LG24	Lca231	DQ290112	GGCCAGGTTAATCAAGAC	ACTAGACTGCAATCAAACACA	55	3

Ta: annealing temperature for PCR.

**Table 2:** Screening of the BAC library with 15 genes/ESTs of *L. calcarifer*

Locus	GenBank accession no	Primer (5'-3', forward)	Primer (5'-3', reverse)	PCR Ta (°C)	PCR product length (bp)	Positive clone number
PVALB-1	AY688372	ATCGTCCGTCCGTTTCCCATAAAA	TGACCTTTCACCTCCCTCCAGACC	55	261	5
5-HT	EU136181	CTGCTCGGCGCGCTCAT	TCCATCCTGCACCTGTGCG	60	200	8
PROL-A	EU136180	GTGCAGAGCCGTCSCCATCA	TTCAGGAAGCTGTCRATCTTGTG	55	500	5
14KDA-AP	EU136179	CCGGGGACAGACAACTCGCTTTCAGAGA	ACAGGTTGGTGAGCTCCAGTTGGTGTTC	55	500	4
AMY-A	AY007592	GGTCGCTTTCCGTAATGTGGTCAA	ACCGGGCATGCCAGTGTTCA	55	250	9
MX	Ay821518	TCATTGATAAAGTGACAGCATTCA	CCAATATCCTTGAGTTTCTTGACA	55	400	7
AP	AJ888375	GACGCCCTCCTCTCCTCTCA	TTTCGACAGCCATCTCTGAACATA	55	700	4
LECT2	EU136177	TTTTTGATCTGAAGATGAGACGTGTCATC	GATCAGATCCCGAGCAGGTCAATC	55	1000	3
LYSO-G	EU136178	AGAGTCCAGGGCTGGAAAT	CCCTCAGAAACTTTAGTTGTGAAC	55	600	9
IGF-1	EU136176	CAGTGGCATTTATGTGATGTC	CCTCGACTTGAGTTTTTCTG	55	503	3
TUB1A	EU136175	GGCACTACACAATCGGCAAAGAGA	TCAGCAGGGAGGTAAAGCCAGAGC	55	144	11
TUB2B	EU136174	GTACAGACGGGGGAAGGGGACCAT	TTCCGCACCCTCAAACTCACCACA	55	160	13
GT7	EU136172	CAGGGTGATCACGCAGTGC	GGCAATCCGACAGCCAGAG	55	156	6
CYP19A2	AY684259	GCTCACCGCCTATAGCCAAAGAA	GGCCGAGTCCTGCCAAGAAA	55	505	8
AFPII	EU136173	TCCCTCCTGTGAAATTGGTTGG	AGGGACGCTGGCACAGACTG	57	1500	14

Ta: annealing temperature for PCR.

### Microsatellite isolation from BAC clones and linkage mapping

**Figure 4:**
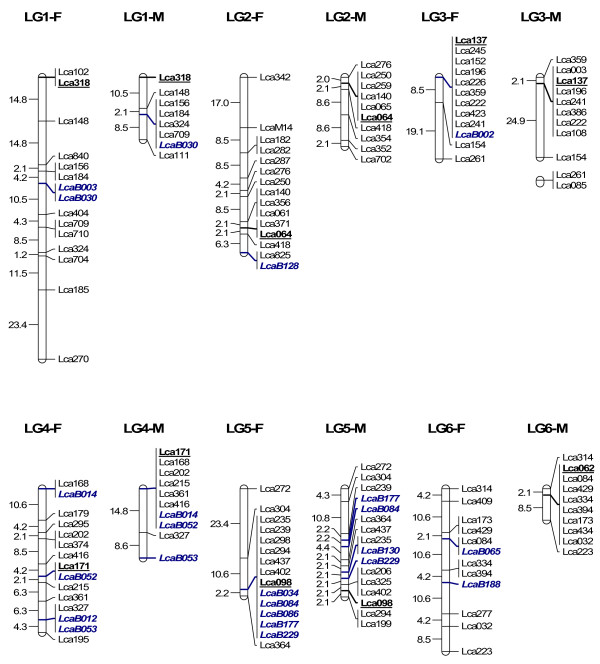
**A microsatellite linkage map of *L. calcarifer *anchored by 86 BAC clones-LG 1–6**. F: linkage map for female. M: linkage map for male. Markers underlined represent microsatellites selected from each LG for screening the BAC library. Markers in italic (initiated with LcaB) represent microsatellites isolated from BAC clones and newly mapped to the map.

**Figure 5:**
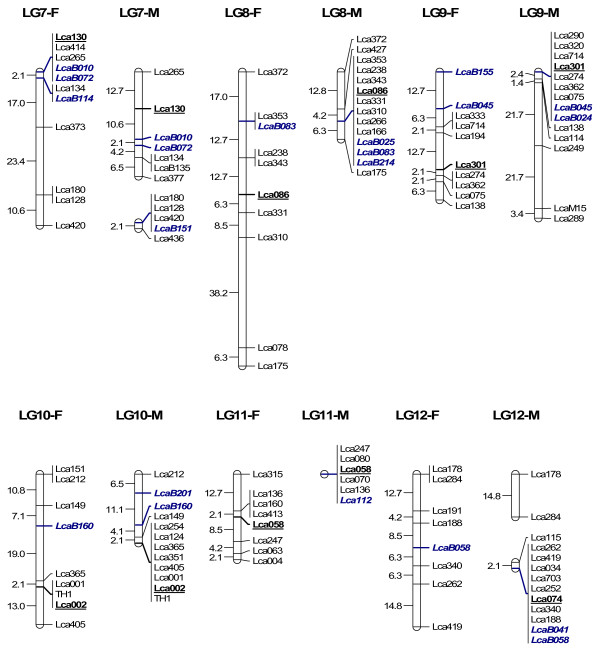
**A microsatellite linkage map of *L. calcarifer *anchored by 86 BAC clones-LG 7–12**. See detailed explanation in Figure 4

**Figure 6:**
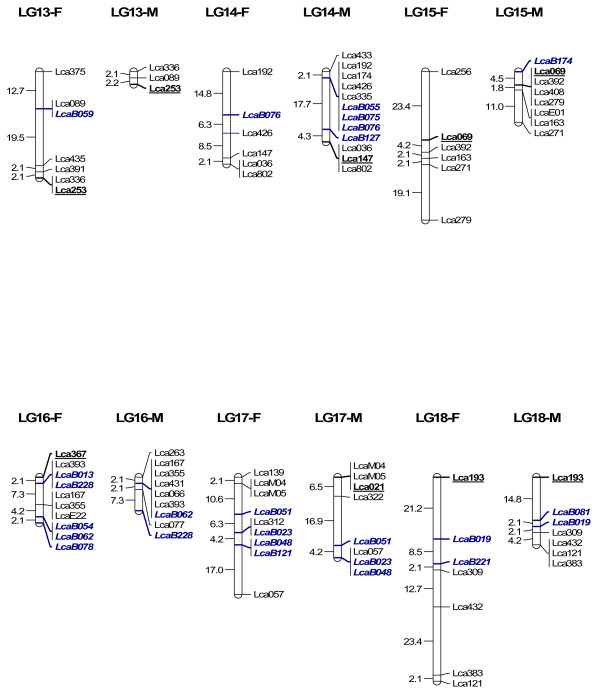
**A microsatellite linkage map of *L. calcarifer *anchored by 86 BAC clones-LG 13–18**. See detailed explanation in Figure 4

**Figure 7:**
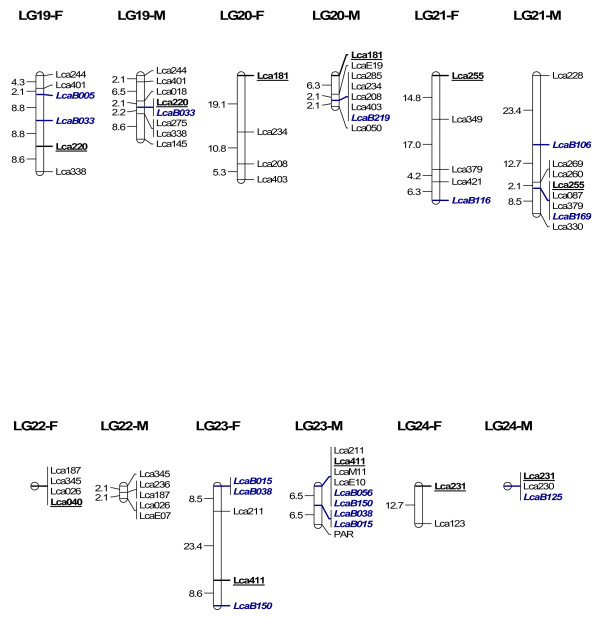
**A microsatellite linkage map of *L. calcarifer *anchored by 86 BAC clones-LG 19–24**. See detailed explanation in Figure 4

In order to map BAC clones to the linkage map for future integrating linkage and physical maps, we isolated microsatellites from 300 BAC clones through enrichment of microsatellites. 864 clones collected from the microsatellite-enriched library were sequenced in both directions. 451 clones contained microsatellites (CA > 7 or GA > 7), yielding 229 unique sequences containing microsatellites. Among the 229 sequences, 218 had enough flanking regions for primer design. Primers were designed for 218 microsatellites, among which 63 within 63 BAC clones were informative in the mapping panel used for linkage mapping. 62 were mapped to 24 LG (Figure 4, 5, 6 and 7) while one marker LcaB044 remained unlinked, making the total number of mapped markers on the *L. calcarifer *linkage map to be 302. At least one BAC based marker was mapped on each LG. Thus, together with 24 microsatellites located in different BAC clones and used for screening the BAC library, a total of 86 BAC based markers have been anchored on the linkage map with at least one on each LG. The male maps of LG14 and LG18, which were split to two LG on the first generation map respectively [6] due to the limited number of markers, were merged to one LG respectively by integrating new microsatellites located in single BAC clones (Figure 6). Details about primer sequences, GenBank accession number, annealing temperature for PCR, PCR product size and locations of the 63 markers located in different BAC clones are summarized in Table 3.

**Table 3:** Microsatellites isolated from BAC clones and mapped on the linkage map of *L. calcarifer*

Orginal order	Linkage group	Locus	GenBank accession no	Motif	Forward primer (5'-3')	Reverse primer (5'-3')	PCR Ta (°C)	Product Length (bp)	Location in the library (384plate-96plate-well)*
1	LG1	LcaB003	EU072356	(GT)_14_	CCTCATACTTGCATCAACATAATA	ATCAAAACCGGCTTCATCT	55	113	128-P2-A3
2	LG1	LcaB030	EU072367	(CA)_27_	TTCTCCCCGTGCCCCTTTGTA	AGCCCACTCCCCTGAGATGAGC	55	158	128-P2-B8
3	LG2	LcaB128	EU072400	(GT)_16_	AGTCGGCCTGTGCAATAAGAT	CAGCAGTTTGGGAATAATGACATA	55	262	128-P1-E4
4	LG3	LcaB002	EU072355	(GT)_10_	TTGGCTGTATTCCTCCTGTCTTGT	TTGGCTCTTTACGCTCAATACTCA	55	182	128-P2-A4
5	LG4	LcaB012	EU072359	(CA)_9_	GTGGGGTGTCCTGGCTCCTC	TCCCATCTCCTCCTGCTGTTTCT	55	329	128-P2-B11
6	LG4	LcaB014	EU072361	(GT)_16_	GCAGACCCGCTTTTTATTCAT	GTCCCCTCTGCTCCAGTGTT	55	181	128-P2-C12
7	LG4	LcaB052	EU072376	(CA)_10_	ATCATGACCCACAAGAGGAGAG	TCAGGGATAGAGACTTGTGAATGA	55	146	128-P2-A5
8	LG4	LcaB053	EU072377	(GT)_18_	GAGGCCCCGATGAGAAAACCTG	TGATGTCGGCGGAGGAGTGC	55	319	128-P2-H11
9	LG5	LcaB034	EU072369	(CA)_8_	TTTGCCTGAATAAAACCCTACACT	AAGCCCTTTGCACAGTATTATTTC	55	171	128-P2-C8
10	LG5	LcaB084	EU072391	(GT)_14_	GAGCGCTCGGCTGTTTCATC	CAGCCAATCTGTTTACCAGCACAC	55	248	128-P2-C4
11	LG5	LcaB086	EU072392	(CA)_12_	CAGATGATCTTTGACGAACTGAAA	TTCTTGGTGAAAAATGACAACAAA	55	157	128-P3-C5
12	LG5	LcaB130	EU072401	(CA)_26_	GGGGGAAAGGAAAAACTGATG	TGTAATGGTAAGATTTTGGGTGTG	55	215	128-P2-F6
13	LG5	LcaB177	EU072409	(GA)_13_	TTTAATTTTAGCCCCGTGATT	GTGTGCCAGTGGGTTTCTC	55	214	128-P3-C1
14	LG5	LcaB180	EU072410	(TC)_13_	AGTCTACACCGATTACACCAGTCT	ACTCTAACCGCACCAGAAAAG	55	243	128-P2-C7
15	LG5	LcaB229	EU072417	(GT)_14_	ACATCGCGTTCTCCTCTGAT	CCAGGGTGTGGTAGTCCTCTC	55	140	128-P3-C8
16	LG6	LcaB065	EU072384	(GT)_13_	GCATTGTTGGCAAAGTTGAGTAT	TCTTACAGTGGGCATCTGACCT	55	148	128-P3-G1
17	LG6	LcaB188	EU072411	(GA)_17_	TGATTTGGCTTTTAGGTGAAACA	TGACAAAAGAATGCCTTGCTCT	55	211	128-P3-D7
18	LG7	LcaB010	EU072358	(CA)_9_	TCCTCCTGGGCTGTTGTATCTTAT	ATGGGGTGGACCTCATTTTCA	55	155	128-P1-G10
19	LG7	LcaB072	EU072385	(GT)_10_	CAACGTGGGTGAATCTGTGT	TTGGCAGCAAATAATTCAGAGTAT	55	217	128-P1-A11
20	LG7	LcaB114	EU072395	(AC)_8_	TGTGCCCATGTTTACTAGATACCA	GTGTGCACGCTGCATTTGT	55	172	128-P2-F9
21	LG7	LcaB135	EU072402	(TC)_18_	CATCCCAGGTTTTCATACCATT	ACTGCGGTTATTAATCCACAAAG	55	123	128-P3-C4
22	LG7	LcaB151	EU072404	(TC)_11_	TTGTGCGCTTCTGTTTGTTTTTCT	GTAGGGCTATGCTGTTGGCTTTCT	55	311	128-P2-D2
23	LG8	LcaB025	EU072366	(GT)_13_	AGGGGGCAAAGGGGTCACG	GAGCCGGCAGTTGCACATCTG	55	160	128-P2-B3
24	LG8	LcaB083	EU072390	(GT)_12_	CGCTGGCATGGCTCTAGTAGTGAT	AGCGGGCTAAAAGCTGCTGTG	55	366	128-P1-H5
25	LG8	LcaB214	EU072413	(GA)_12_	AGCGGGAGGCTGAGAAGTAA	ACCCCTGCCTCTTGTTCATC	55	239	128-P1-H4
26	LG9	LcaB024	EU072365	(GT)_10_	AGAAGGGAAAAAGAGATGGGATGT	CAGGGCCGTTTTATTGCTGTAG	55	162	128-P3-B2
27	LG9	LcaB045	EU072373	(GT)_26_	ACAGGGAACGAATGGGGACAA	AAATTGGCACGCTCATTCAAGAAC	55	149	128-P2-D4
28	LG9	LcaB155	EU072405	(GA)_24_	TGTGGCCTTTGTGTAAGTGAGAA	TCATTCCCGCAAACAACACA	55	197	128-P3-G11
29	LG10	LcaB160	EU072406	(GT)_13_	CTTCATCCAGCCCAGTGACAG	GAATGGCCAGCTAAAACATCAAC	55	307	128-P3-A1
30	LG10	LcaB201	EU072412	(TC)_16_	ATTGCACCAGTCCCGAATGAG	GCAGCGTGCTTGTGGAAAAA	55	210	128-P2-D1
31	LG11	LcaB112	EU072394	(GT)_7_	TACCTGCCTTGTTTTTGTCCTTA	AAGCCTCCATACACAGCTACATTA	55	113	128-P1-D6
32	LG12	LcaB041	EU072371	(AC)_8_	AGGTATGTTTTTGGGGCTTTTAGT	CCCCCTACCCCTGTTTTACATA	55	250	128-P1-B5
33	LG12	LcaB058	EU072381	(AC)_15_	AAACCAAATGCTTACACAGTTACC	TTGAGAGCTATTGGGATTACACAT	55	160	128-P1-A2
34	LG13	LcaB059	EU072382	(AC)_18_	CCTAGCCAAGTGCAACAGTGTG	AGCTGGGAAACAGGCTGAGAC	55	186	128-P3-A12
35	LG14	LcaB055	EU072379	(AC)_12_	AGTTGCGGTCTTGTCCAAATGG	ACTGGCAGAGTCAAGCAAAGTGTG	55	325	128-P3-A3
36	LG14	LcaB075	EU072386	(GT)_12_	TGTCGCACACCGCTGCTTTACTAT	CTTGCTCTCACCCTCTCCCTCTTT	55	131	128-P2-G12
37	LG14	LcaB076	EU072387	(AC)_17_	CGAAAACGTCGATCCAACTAAA	ACAGTCAGTGCGTGAAGTGTATG	55	135	128-P3-A2
38	LG14	LcaB127	EU072399	(AC)_11_	AGTTGCAGGGCATGCTGTGAAAC	TCGGCATCAAGCGTGGAAGAG	50	159	128-P2-D6
39	LG15	LcaB174	EU072408	(GT)_8_(GA)_15_	CAGCATTAAAAAGATGAGAAAAGT	ATTCCCCCATCTTTGTTACAGTT	55	242	128-P2-D7
40	LG16	LcaB013	EU072360	(AC)_15_	AGGCCAAGGCTGCTCTGTGTC	CAACCTGGGATGAGGCACTAAAAG	55	127	128-P2-B12
41	LG16	LcaB054	EU072378	(AC)_8_	TGCAGGAGATAAGACGCTGTG	GAGATCGGCAACCTGACAAA	55	298	128-P3-F4
42	LG16	LcaB062	EU072383	(AC)_15_	ATGAGGGGTGAACAGTTGTCCT	TCTCCTCGTCCTTTTCGTTACC	55	218	128-P3-F8
43	LG16	LcaB078	EU072388	(GT)_13_	GTTACCATGCCAACAACCAA	TAGCCTGCTATAGATCCCACTG	55	81	128-P3-A4
44	LG16	LcaB228	EU072416	(AC)_21_	GAATAGGCCTACCTGGTGAGAGG	TCCCTGCTTAGCTGCCATTATC	55	237	128-P2-B12
45	LG17	LcaB023	EU072364	(GT)_13_	GCAGCGAGATGAACAGTGATTATT	ACATGATCCTCGCCACCATC	55	326	128-P2-G5
46	LG17	LcaB048	EU072374	(GT)_20_	TGGAGCTTTATTTGAGTGTGAC	CCCCCTATGTATTCAGTATTCTG	55	180	128-P3-C4
47	LG17	LcaB051	EU072375	(GT)_19_	TACCCAAAGTAAACCAGCAGCACA	CAACTAGCAGGTTTGCACAACACA	55	104	128-P3-H11
48	LG17	LcaB121	EU072397	(AC)_16_	CTTTTTGTGCCCCAGATGACG	GGAGCAGAGTGGAGCTTTCAGAA	55	238	128-P1-D9
49	LG18	LcaB019	EU072363	(GT)_11_	TTGAGTCCCCTGTGCTATGTAACA	CACCGCCTCCACAATTAGTGTC	55	199	128-P1-F10
50	LG18	LcaB081	EU072389	(GT)_7_(GCA) (GT)_3_	TGAGGACAGCCACCCCACTTTT	GAGCCGCTATCTCATTCCCACATC	55	126	128-P2-F10
51	LG18	LcaB221	EU072415	(TC)_9_	AGGGGAGTGCTGCCTCAGTG	TTCCCAACAGATAATGATGCTCAA	55	117	128-P3-A8
52	LG19	LcaB005	EU072357	(AC)_22_	AGGCGGTGCTGGGGCAGAT	TTACCGCAGCCTGGCTAGAGGTCT	55	300	128-P3-H8
53	LG19	LcaB033	EU072368	(AC)_15_	ATCCACCTTGAGGTTTCTTTATCA	AACCAAGCCACTCCTATCATCTT	55	190	128-P1-D5
54	LG20	LcaB219	EU072414	(GA)_25_	AGTTGGCTCTTAAAGCATTTGAAT	TTCCCACACCGTTAGGTTTATCTG	55	155	128-P1-H12
55	LG21	LcaB106	EU072393	(GT)_7_	CTGGCTGCATGGAGAAAGAAGT	TTGGGTTTTGAGCTCACTGACA	55	311	128-P2-F7
56	LG21	LcaB116	EU072396	(GT)_20_	CATGGCCTTTCTGGGAAGTTATTG	CAGACGGAGCCACAAGCAAAAC	55	226	128-P3-D6
57	LG21	LcaB169	EU072407	(AC)_6_(GA)_20_	CACAAACCAGGCGATCACATATCG	GTAAGCCCGCAGAAATCGACTTCA	55	218	128-P3-E9
58	LG23	LcaB015	EU072362	(GT)_11_	GAGCGCTCTCCCCTGGTTTC	TGCAGCCGAGCACGACTG	55	221	128-P1-G9
59	LG23	LcaB038	EU072370	(GT)_19_	TGTGCGCACTCACATACATTAG	TGAAAAATAGATGGTAAGCCTCTC	55	216	128-P1-A3
60	LG23	LcaB056	EU072380	(AC)_11_	ATGCCGTTTCCTGCTGCTGTC	TGATGCTGTTTCTGGCTGGTGTA	55	141	128-P2-E02
61	LG23	LcaB150	EU072403	(GA)_11_	TCTAGCGCTCGTCCTCTCCTG	AGGCCTCCTCGTTCTCTGCT	55	178	128-P2-A11
62	LG24	LcaB125	EU072398	(GT)_12_	AAGCACAAGATACGCCTTCCTT	GTGCCCTGGGCCTCTACAT	55	153	128-P2-C11
63	Unlinked	LcaB044	EU072372	(GT)_15_	CAGGACGTTTGAATACTTGTGT	TTAAAAGGTGGTGGTATTAGTCAT	55	160	128-P2-A8

Ta: annealing temperature for PCR. * 384plate-96plate-well: name of the 384-well plate, name of 96-well plate and well position in the 96-well plate.

## Discussion

A critical tool for genomic studies in fish species is the availability of deep-coverage large-insert genomic libraries, such as BAC libraries that can be used for physical mapping, integration of linkage and physical maps, positional cloning, comparative genomic studies and genome sequencing [13]. We constructed the first BAC library for *L. calcarifer *containing 49,152 clones with an average insert size of 98 kb ranging from 45 to 200 kb, indicating that this BAC library provided 6.9 × coverage of the *L. calcarifer *haploid genome. We have noticed that 50% of the inserts in our BAC library were under 100 kb. It is common that insert size of 50% of BAC clones was smaller than the size of DNA fragments recovered from gels. This phenomenon has been seen in several BAC libraries, such as the BAC library of tomato [13]. The reason for this is that smaller fragments could be included in larger fragments during electrophoresis, and during ligation, the relatively smaller fragments were preferentially ligated to the vectors. PCR screening of the library with 24 markers each from one of 24 LG and 15 randomly selected ESTs/genes demonstrated that the BAC library provided good coverage of the *L. calcarifer *genome. Whether the BAC clones with large inserts were of hybrid origin remains to be examined.

A second generation linkage map of *L. calcarifer *is under construction by integrating new markers including microsatellites, ESTs and candidate genes onto the first generation map. Low polymorphism of ESTs and candidate genes was a bottleneck to map them to the linkage map [6]. Using highly polymorphic microsatellites located in BAC clones harboring interesting genes and ESTs, these interesting genes and ESTs could be mapped onto the linkage map as shown in this experiment. By employing highly polymorphic microsatellites in BAC clones, we have mapped 86 BAC clones to the linkage map of *L. calcarifer*. At least one BAC clone has been anchored on each LG, which can be used to integrate linkage and physical maps in the future. The number of markers on the linkage map of *L. calcarifer *increased to 302 by mapping 62 novel microsatellites located in 62 BAC clones onto the map. The two male linkage groups (i.e. LG14 and LG18) which were split to two LG on the first generation map respectively [6] due to the limited number of markers on these LG, were merged to one LG respectively by integrating new microsatellites located in single BAC clones, which improved the quality of the linkage map of *L. calcarifer*.

The BAC library of *L. calcarifer *could be also used in constructing a physical map by BAC fingerprinting [12,20], sequencing BAC ends and positional cloning of QTL of commercial interests [4] so as to facilitate selective breeding of *L. calcarifer*. Eventually, the BAC library can be used in whole genome shotgun sequencing when it becomes necessary.

## Conclusion

A first *L. calcarifer *BAC library with 6.9 × coverage of the haploid genome has been constructed and characterized. Screening the library with 24 markers and 15 ESTs/genes demonstrated good genome coverage of this library. Eighty-six BAC clones were mapped to the first generation linkage map, improving the marker density of the linkage map of *L. calcarifer*. This BAC library together with the improved linkage map not only supplies an indispensable tool to physical mapping, integration of physical and linkage maps, and positional cloning for genes of importance, but also contributes to comparative genomic studies and eventually genome sequencing.

## Methods

### Preparation of high-molecular-weight DNA

Five hundred microliters of blood was collected from a male individual of *L. calcarifer *with a heparinized syringe. The concentration of leucocytes was quantified to be approximately 10^9 ^cells/ml. Quantities corresponding to 2.14 × 10^7 ^cells were embedded in 40 μl of 2% InCert agarose (in PBS) for DNA extraction. The mixture was then transferred into ice-cold plug moulds (Bio-Rad, SG, Singapore). Individual plugs were released into cell lysis solution [1% lithiumdodecyl sulfate, 10 mM Tris (pH 8), 100 mM EDTA (pH 8)] that was incubated at 37°C for 1 hr with occasional swirling. The cell lysis solution was replaced with 50 ml of new cell lysis solution and incubated overnight at 37°C. The cell lysis solution was supplanted with 50 ml of 20% NDS. Two ml of proteinase K (20 mg/ml) was added to each 50 ml of 20% NDS consisting of 0.2% N-laurylsarcosine, 2 mM Tris-HCL (pH9.0), 0.14 M EDTA. The solution was incubated at 37°C overnight. Plugs were washed three times with TE50 and 0.05 M EDTA for one hour at room temperature. The plugs were put into a fresh Falcon tube, and washed twice with 50 ml TE50 and 50 μl PMSF (100 mM) at 37°C for 20 min to inactivate proteinase K. The plugs were then washed twice with 50 ml of TE50 in the Falcon tube at room temperature for 30 min to get rid of the PMSF.

### Partial digestion of high molecular weight DNA and size selection

Digestion with restriction enzyme *Hin*dIII, pulse field gel electrophoresis (PFGE), isolation and purification of high molecular weight (HMW) DNA were performed using the method described previously [21]. Briefly, after displacement of the plugs by 1 × TE buffer, the agarose plugs were soaked in 800 μl of *Hin*dIII digestion buffer [0.015% bovine serum albumin (BSA), 75 mM NaCl, 12 mM Tris-HCl (pH 7.50)] and 3 U of *Hin*dIII for 16 hours at 4°C, after which, 100 μl of MgCl_2 _(100 mM) was added and the mixture was incubated at 37°C for one hour to partially digest the genomic DNA. The reaction was stopped by adding 150 μl of 0.5 M EDTA (pH 8.0), 15 μl 20 mg/ml proteinase K and 37.5 μl 20% NDS, and incubating at 37°C for one hour. Plugs were rinsed in TE50 in a Petri dish then transferred to a 15 ml Falcon tube. 15 ml of TE50 and 15 μl of 100 mM PMSF were added to the tube. The tube was incubated at room temperature for 20 min on rotating shaker. The tube with plug was washed twice with 15 ml TE50 at room temperature on a shaker for 30 min each.

Size selection was carried out as described [22], with minor modifications. In brief, partially digested DNA was separated by PFGE in 0.5 × TBE on a CHEF-DRII apparatus (Bio-Rad, SG, Singapore) under the following conditions: 14°C, 6.0 v/cm, angle = 120°, initial switch time = 5 sec, final switch time = 15 sec, run time = 16 hours and ramping = linear. At the end of this electrophoresis step, the gel portion containing DNA of 50 kb or less in size together with the portion of the gel containing the original plugs was removed. 1% fresh agarose was added to the remaining gel followed by a second electrophoresis step using the same conditions for 18 hours. Gel slices containing size fractionated DNA were obtained by cutting horizontally at 0.5 cm intervals in the size range of 100–250 kb. Each excised gel slice was subsequently inverted and buried in 1% low-melting-point agarose gel. A third electrophoresis step using the same conditions for 18 hours was carried out to concentrate the widely spread DNA fragments in each gel slice into a sharp single band. The band of size selected genomic DNA was then excised and dialyzed in 1 × TAE at 4°C overnight.

### Ligation and electroporation

Size fractionated DNA was recovered from each gel band by electroelution in Spectra/Por 7 dialysis bags (Spectrum Laboratories, CA, USA) as described [23]. Partially digested HMW DNA was then ligated to 25 ng of dephosphorylated, *Hin*dIII digested pCC1BAC (Epicentre, MD, USA) at a 1:10 molar ratio of insert to vector with 400 units of T4 ligase (NEB, MA, USA) in 50 μl reaction at 16°C overnight. Dialyzed ligation was used to transform ElectroMAX DH10B competent cells (Invitrogen, MD, USA). Electroporation was carried out using a BioRad Gene Pulser (BioRad, CA, USA) at 4 kΩ and 350 V. Cells were incubated in 1 ml SOC medium at 37°C for one hour with shaking and later spread on LB plates containing 12.5 μg/ml Chloramphenicol, 40 μg/ml X-gal and 100 μg/ml IPTG and incubated at 37°C for 24 hours to allow the blue color to develop sufficiently.

### Isolation of BAC DNA and estimation of insert size

We isolated BAC DNA from 212 BAC clones randomly chosen using a QIAwell 8 Plasmid Kit (Qiagen, HRB, Germany) following the protocol of the manufacturer. Isolated BAC DNA were digested with the restriction enzyme *Not*I and then subjected to PFGE for 16 hours using the same PFGE conditions as those for high molecular weight DNA isolation.

### Library pooling and PCR screening

White recombinant colonies were manually picked and arrayed to plates (Genetix, Hampshire, UK) of 384-well each containing of 60 μl of LB media and 25% glycerol. Plates were incubated overnight at 37°C and stored at -80°C. The frozen stocks of the primary clones in 384 well plates were recovered and transferred to 4 96-well PCR plates containing 100 μl LB medium supplemented with 15% glycerol and 12.5 μg/ml chloramphenicol, then incubated overnight at 37°C to make a copy of the BAC library.

To establish a hierarchical PCR screening system, the library was divided into 11 superpools each consisting of 12 plates of 384-wells. Each superpool was divided into 48 pools each consisting of one 96-well plate of BAC clones. Cultures from 48 pools were combined to make superpool DNA for the first step PCR screening. Cultures from 48 plates of 96-well BAC clones were combined to make pool DNA for the second step PCR screening. In each pool, cultures from each well of 96 clones from a 96-well plate were used for the third step screening.

For examining the genome coverage of the BAC library, twenty-four microsatellites (Lca318, Lca064, Lca137, Lca171, Lca098, Lca062, Lca130, Lca086, Lca301, Lca002, Lca058, Lca074, Lca253, Lca147, Lca069, Lca367, Lca021, Lca193, Lca220, Lca181, Lca255, Lca040, Lca411 and Lca231) located on each of the 24 linkage groups (Table 1) [6], and 15 ESTs/genes isolated from cDNA libraries or selected from GenBank were used to screen the library. These 15 ESTs/genes are: PVALB-1, 5-HT, PROL-A, 14KDA-AP, AMY-A, MX, AP, LECT2, LYSO-G, IGF-1, TUB1A, TUB2B, GT7, CYP19A2 and AFPII. Primers (Table 2) were designed in unique regions for each EST/gene using software PrimerSelect (Dnastar, WI, USA). The PCR reaction (25 μl) consisted of 2 μl cultured cells, 1 × PCR buffer (Finnzymes, Espoo, Finland) containing 1.5 mM MgCl_2_, 200 nM of each primer, 50 μM of each dNTP and one unit DNA polymerase (Finnzymes, Espoo, Finland). PCR was conducted on a PTC-100 PCR machine (MJ Research, CA, USA) using the following PCR program: an initial denaturation at 95°C for 2 min followed by 35 cycles 95°C for 30 sec, 55°C for 30 sec and 72°C for 1–2 min, and a final extension at 72°C for 5 min. PCR products are checked for the presence of PCR products on 2% agarose gels. Positive pools were used to determine a set of addresses corresponding to potential clones, which were subsequently validated by a third PCR analysis of individual clones. PCR products of respective microsatellites and genes/ESTs were confirmed by direct sequencing.

### Microsatellite isolation from BAC clones and linkage mapping

DNA was isolated from pool of 300 BAC clones using a QIAwell 8 Plasmid Kit (Qiagen, HRB, Germany). CA- and GA-microsatellites located in the 300 BAC clones were enriched according to a previous protocol [24] with some modifications [25]. Repeat-enriched DNA fragments of 400–1200 bp in size were cloned into pGEM-T vector (Promega, CA, USA), and transformed into XL-10 blue supercompetent cells (Stratagene, CA, USA). White clones were picked and arrayed into 96-well plates for bidirectional sequencing on an ABI3730 × l DNA sequencer (ABI, CA, USA) using the BigDye V3.0 kit, M13 forward and M13 reverse primers. Redundant and overlapping sequences were grouped using Sequencher (GeneCodes, MI, USA). Unique sequences were compared to known microsatellite sequences of *L. calcarifer *prior to primer design in order to reduce redundancy. Genotyping and linkage mapping of these microsatellites were performed with the mapping panel described previously [6]. The graphic maps were generated using Mapchart software [26]. To identify the origin of each microsatellite from the 300 BAC clones, these clones were PCR-screened with microsatellite primers. PCR products were checked for the presence of objective bands on 2% agarose gels.

## List of abbreviations

BAC-bacterial artificial chromosome; QTL-quantitative trait loci; LG-linkage group; PVALB-1-pavalbumin beta gene 1; 5-HT-5-hydroxytryptamine type 1 receptor; PROL-A-prolactin gene alpha type; 14KDA-AP-14kDa apolipoprotein gene; AMY-A-amylayse gene alpha type; AP-aminopeptidase gene; LYSO-G-lysozyme goose type; TUB1A-tublin 1 alpha type; TUB2B-tublin 2 beta type; GT7-EST containing a (GT)_7 _microsatellite, CYP19A2-cytochrome P450 aromatase alpha type 2 and AFPII-type II antifreeze protein.

## Authors' contributions

GHY planned and started the project, and determined the final version of the manuscript. CMW designed and conducted the experiment, as well as drafted the manuscript. LLC, FF, GP, LJ, ZZY and LG are involved in screening the library with randomly selected microsatellites and genes, mapping of markers to the linkage map. All authors have read and approved the final version of the manuscript.
